# Changes of Routine Hematological Parameters in COVID-19 Patients: Correlation with Imaging Findings, RT-PCR and Outcome

**DOI:** 10.30699/IJP.2021.533645.2675

**Published:** 2021-12-15

**Authors:** Abdolreza Javadi, Shahriar Dabiri, Manzumeh Shamsi Meymandi, Mohammad Hashemi Bahremani, Hussein Soleimantabar, Bahram Dabiri, Houman Vosough, Maryam Gheidi Sharan, Farnoosh Sedaghati

**Affiliations:** 1 *Department of Pathology and Laboratory Medicine, Imam Hossein Hospital, Shahid Beheshti University of Medical Sciences, Tehran, Iran*; 2 *Imam Hossein Central Medical Laboratory, Shahid Beheshti University of Medical Sciences, Tehran, Iran*; 3 *Department of Pathology, Afzalipour Medical School, Kerman, Iran*; 4 *Pathology and Stem Cells Research Center, Kerman Medical School, Kerman University of Medical Sciences, Kerman, Iran*; 5 *Department of Radiology, Imam Hossein Hospital, Shahid Beheshti University of Medical Sciences, Tehran, Iran*; 6 *Department of Pathology, Resident NYU Langone Health, Mineloa, NY, USA*; 7 *Department of Hematology, Iran University of Medical Sciences, Tehran, Iran*

**Keywords:** COVID-19, Hematology, Tomography scanners, Viral load

## Abstract

**Background & Objective:**

Coronavirus disease 2019 (COVID-19) is progressively spreading, and many researchers have focused on the prognostic value of laboratory analyses. This study reviewed routine blood parameters, upper respiratory viral load, and chest imaging in recovered and expired COVID-19 patients and evaluated possible correlations.

**Methods:**

In this retrograde study, 138 COVID-19 cases were enrolled. Chest tomography scores of patients, routine hematologic and biochemical parameters, and respiratory viral loads were measured. Furthermore, their correlation with severity of disease and the outcome was investigated during a week of admission.

**Results:**

The mean age of participants was 58.6±16; 36.2% of whom were diagnosed as critical, 8.7% expired, and 46% showed less than 50% lung opacity. The expiring rate was only correlated to the severity of illness and viral load. During admission, hemoglobin concentration was decreased in critical patients (from 11.49±0.27 to 10.59±0.36, *P*=0.042) and also among CT-scan scoring groups (*P*=0.000), while neutrophils (*P*=0.04), WBC (*P*=0.03), and platelets (*P*=0.000) count were increased. In patients with more than 50% lung opacity, leukocyte counts were decreased, but neutrophil and platelets counts showed raise (all *P*<0.05), while other hematologic parameters did not change. CRP and LDH demonstrated no increase based on the severity of the illness, RT-PCR viral loads and/or outcome. However, both CRP and LDH were increased in patients with more than 50% lobal opacity (CRP: 69.3±9.9 to 1021.1±7.5 and LDH:589.5±93.2 to 1128.6±15.81,* P*<0.05).

**Conclusion:**

We found that hemoglobin, white blood cells, neutrophil, lymphocytes, and platelets count together with chest tomography score might be beneficial for expedition the diagnosis, assessmen the severity of the disease, and outcome in the hospitalized cases, while CRP and LDH might be considered as the consequence of lung involvement.

## Introduction

An outbreak of Coronavirus disease 2019 (COVID-19) was reported in December 2019 in Wuhan, China (1, 2). It rapidly became a pandemic that infected more than 206 million people worldwide so far .The death toll has been reported 4.35 million until August 14, 2021, in 191 countries and regions. The first case in Iran was confirmed on February 19, and by mid-August 2021, 4.36 million laboratory-confirmed cases had been documented in Iran (3). Given that some patients only show a mild course of disease with a good prognosis and others suffer greatly with complex treatment and high mortality, classification of the severity of disease is crucial to guide the proper treatment and care (4).

More researches are needed to reveal the risk factors such as laboratory data and biomarkers for severe and critical COVID-19 patients. It was previously emphasized that laboratory medicine plays a vital role in the early detection, diagnosis, and management of many diseases (5, 6). Various hematological parameters are currently being utilized to predict outcomes, mortality and guide treatment in patients infected with SARS-COV-2 (5).

In two systematic reviews of 727 articles most frequent laboratory abnormalities in COVID-19 patients were the decreased albumin, high C-reactive protein (CRP), elevated lactate dehydrogenase (LDH), lymphopenia, and high erythrocyte sedimentation rate (7, 8). A retrospective study of routine blood analyses from 137 confirmed COVID-19 critical cases admitted to the University Hospital of Leuven in Belgium reported significant lower platelets, eosinophils, lymphocytes, and monocytes with higher leukocytes and neutrophils count compared to recovered patients. The difference in lymphocytes count showed substantial results. These hematological cytopenias were signi-ficantly lower between day 9^th^ and 12^th^ after admission making this time window important in predicting clinical worsening of a patient (9). A study of 1641 admitted patients having COVID-19 detected through PCR presumed that the number of platelets and their dynamic changes during the treatment may suggest the severity and prognosis of the disease. The patient with markedly elevated platelets and longer average hospitalization days may be related to the cytokine storm (10). 

Published research revealed that blood cell count analysis is a simple, cost-effective, and rapid laboratory diagnostic tool for evaluating infectious inflammatory responses and the severity of COVID-19. Higher leukocyte and neutrophil count while lower lympho-cytes, red blood cell count, hemoglobin concentration, and hematocrit levels were found in critical patients at the end of treatment (11). Also, a cross-sectional study of 148 patients showed older age, neutrophilia, lymphopenia, eosinopenia, high neutrophil-lymphocyte ratio, and high neutrophil-monocyte ratio were associated with severe and critical COVID-19 infection. Lymphocyte (%), monocyte (%), and eosinophil (%) were negatively correlated to disease severity (12). A similar study of COVID-19 hematological parameters disclosed that severe and critically ill patients had significantly lower lymphocyte count, decreased red blood cell and hemoglobin compared with regular COVID-19 patients (13). The results of a meta-analysis emphasized that hemoglobin values are essentially reduced in COVID-19 patients with severe and critical diseases compared to those with milder disease (28). 

A comprehensive study on 3014 cases showed the essential role of nasopharyngeal (NP) viral load in SARS-COV-2 pathogenesis and suggested that repor-ting viral load results to clinicians is valuable laboratory data in the care of hospitalized patients with COVID-19 (14). A possible association between NP viral load and serum biomarkers such as CRP and SARS-COV-2 IgM antibodies has been suggested. In IgM reactive patients, oropharyngeal (OP) viral load showed different trends among various cases with different severity (15). In contrast, a reversed correlation between viral load and bilateral chest involvement in chest tomography has been reported (16).

There are limited studies in medical literature discussing the correlation between SARS-COV-2 viral loads, laboratory abnormalities, and disease spectrum. This possible relationship has not been distinctly investigated. 

Our study aims to evaluate routine hematologic abnormalities in confirmed and suspicious COVID-19 patients during the first week of admission in order to reveal the association between imaging (CT-scan), clinical severity, common biomarkers (Q-CRP, LDH), routine complete blood count parameters, and NP/OP viral loads.

## Material and Methods

**Patients**

This is a retrospective study of 138 confirmed and suspicious COVID-19 cases, according to WHO guidelines for the diagnosis of SARS-COV-2 (17), admitted to the Imam Hossein Hospital (Tehran, Iran) between August and September 2020.

Routine confirmation of cases of COVID-19 was based on the detection of unique sequences of virus RNA with real-time polymerase chain reaction (RT-PCR) (17).

The included cases consisted of SARS-COV-2 molecular confirmed patients and suspicious COVID-19 cases admitted to the hospital because of the severity of symptoms according to medical assessment and imaging findings. All molecular analyses were performed on admission to the hospital. Also, low-dose lung CT scans were performed for all patients using a 16 detector CT scan machine (SIEMENS; SOMATOM). Main CT parameters were kvp:100, mAs: 50-100, pitch:1.5, thickness: 4mm. The patterns of lung involvement consisting of ground glass, consolidation, crazy paving, and reverse halo, a form of involvement (such as round, linear and non-specific), distribution, and severity were reviewed by the consultant radiologist. A semi-quantitative chest tomography scoring was applied to estimate the severity of lung parenchymal involvement. The lung lobes CT-scan were visually scored to the following groups: No involvement (with less than 5% involvement), Subtle (from 5 - 25% involvement), Mild (from 26- 49% involvement), Moderate (from 50- 75% involvement), and Severe (more than 75% involvement). Image scoring system was acquired from Pan* et al.*, published article (18). Moreover, according to the published severity classifications, COVID-19 patients were classified into critical (ICU admitted) and non-critical (19). Furthermore, all patients were divided into two groups of less and more than 50% of lung CT-scan involvement.

The clinical laboratory tests, as well as NP/OP RNA viral load, were compared between non-critical and critical (ICU admitted), outcome (recovered or expired), and among CT-scan groups (subtle, mild, moderate, and severe) at the time of admission and after the first week of hospitalization. 

**Data Collection**

Demographic data, imaging findings, and laboratory results of COVID-19 cases were collected from Imam Hossein Hospital LIS and HIS affiliated Beheshti Medical University (Tehran, Iran). The anonymity of the patients was protected. 

**Laboratory Analysis**

NP swab and/or an OP swab are recommended for screening or diagnosis of the infection (20). The NP and OP specimens were obtained from patients at admission time and transported via the viral-transport medium.

A total of 10 μL of extracted RNA was used for molecular analysis. Magcore® HF16 automated nucleic acid extractor (Taiwan) was used for nucleic acid extraction from 400 mL throat swab elute per sample. Reverse transcriptase-polymerase chain reaction (RT-PCR) assay of COVID-19 was confirmed according to the cycle threshold values for ORF1ab- and N-genes. The assay was performed by the core hospital Laboratory based on standard molecular protocols (21, 22). 

Real-time RT-PCR was performed using the following conditions: 45°C for 10 minutes, denaturation 95°C for 2 minutes, 45 cycles of ampli-fication at 95°C for 15 seconds, and 58°C for 45Seconds(RT steps). The tentative limit of detection (LoD) was determined to be 150 copies/mL. The Cycle threshold values were inversely related to viral RNA copy numbers, with a Cycle threshold <33 being considered positive (Euroimmune^® ^EURORealTime SARS-COV-2 MP 2606-0100). Based on the manufacturer manual, (Positive agreement 96.0% ,C.I.: 86.3% to 99.5% and Negative agreement 100.0% C.I.: 88.4% to 100.0%) the assay positive and negative agreements are 96.0% and 100.0%, respectively.

Laboratory analysis, including complete blood count (CBC), C- reactive protein (CRP), lactate dehydrogenase (LDH), are selected for analysis, considering the routine hematological abnormalities in COVID-19 patients (5) and recommendations for COVID-19 laboratory test panels (23). 

EDTA blood samples were collected from patients. Hematology parameters include white blood cell count (WBC), absolute lymphocyte count (LAC), absolute neutrophil count (NAC), monocytes absolute count (MAC), and eosinophils absolute count (EAC) were performed with Siemens ADVIA2120^®^ applying peroxidase flow cytometry method. 

Blood biochemistry tests were assessed using HITACHI 919 and Biolis 24i automated random-access analyzers. All samples were labeled using a barcode tracking system and then analyzed at the hospital core laboratory in a duplicated manner. Commercial Siemens, SERO^®^ QC materials for internal QC, and RANDOX^®^ external quality control programs (RIQAS) were applied for laboratory quality control. 

**Statistical Analysis**

Data were expressed as frequency and mean ± SE and compared using the independent and paired t-test or one-way ANOVA, followed by Tukey post-hoc. Categorical values were analyzed using Fisher’s exact test or χ ^2^ test. All analyses were performed using SPSS 23.0 software (SPSS Inc., Chicago, IL., USA). A two-sided P-value<0.05 was considered statistically significant.

**Study Approval**

The local Research Committee of Beheshti University of Medical Sciences (Pajoohan) accepted the proposal of this study (99-25032), and the Ethical Committee approved it (IR.SBMU.RETECH.REC-.1400.349). No informed consent was acquired because this was a retrospective study**.**

## Results

A total of 138 patients were included in the study. The mean age was 58.6±16 years and 50%, male. From the total number of cases, 8.7% were expired, and 36.2% were diagnosed as critical. The CT-scan of 32% was moderate, and 46% showed less than 50% lung lobe area of opacities. RT-PCR positive rate was 79%. The CT scan scoring was based on hospital imaging reports (Table. 1).

All expired patients were diagnosed as critical, and a significant correlation was found between outcome and illness severity (*P*=0.000), while Imaging rating or SARS-COV-2 RT-PCR was not dependent on diagnosis (Table. 2). However, most of the patients diagnosed as critical showed moderate Image rates and positive RT-PCR (Table 2).

Expiring rate (Outcome) was correlated to RT-PCR (*X*^2^= 5.40, *P*=0.042) since 64% of expired patients showed positive RT-PCR. No correlation was found between expiring rate and Image scoring.

**Table 1:** Clinico-labarotery haracteristics of the 138 COVID-19 patients

Outcome	No. (Percent)
Expired	**12 (8.7%)**
Recovered	**126 (91.3)**
Severity of illness	**No. (Percent)**
Critical	**50 (36.2%)**
Non-critical	**88(63.8%)**
CT-scan groups	**No. (Percent)**
Subtle	**13 (9.4%)**
Mild	**77 (26.1%)**
Moderate	**45 (32.6%)**
Severe	**3 (2.2%)**
No involvement	**41(29.7%)**
RT-PCR	**No. (Percent)**
Positive	**109 (79%)**
Not detected	**17 (63.8%)**
Not provided	**12 (8.7%)**

The descriptive Table of 138 patients referred to Imam Hossein Hospital. The number (no) and respective percent (%) were defined based on the outcome, severity of illness, groups of CT-scan imaging, and viral load of SARS-COV-2.

**Table 2:** Correlation of the outcome, CT-scan score, and RT-PCR rate with the severity of illness

		Critical(no)	Non-critical(no)	P*-*value
Outcome	Recovered	38	88	**0.000**
	Expired	12	0	
CT-scan group	Subtle	4	9	**0.48**
	Mild	9	27	
	Moderate	19	26	
	Severe	1	2	
RT-PCR	Positive	40	69	**0.46**
	Not detected	7	10	

The distribution of the number of Critical and Non-critical patients (severity of illness) between Recovered and Expired patients was significantly different, while among CT-scan groups and between RT-PCR, positive and not detected was not different.

Comparison of routine hematological factors showed no significant difference between the severity of illness groups (critical vs. non-critical). None of the hematologic parameters, including LAC, NAC, EAC, and MAC, changed between groups of critical vs. non-critical, neither between groups of recovered vs. expired (Outcome) or between CT-scan involvement above 50% vs. under 50%. But the mean hemoglobin concentration during admission (Day 0) was lower in critical compared to non-critical patients (from 11.49-±0.27 to 10.59±0.36, *P*=0.042). Hemoglobin concen-tration and C-reactive protein showed significant changes among CT-scan scoring groups (Table 3).

**Table 3:** The mean of significant laboratory analyses based on CT-scan groups

	Subtle	Mild	Moderate	Severe	P-value
Hemoglobin_(g/dl)_ Day 0 Hemoglobin_(g/dl)_ Day 7	9.7±0.8 8.59±0.46	11.21±0.33 10.63±0.35*	12.77±0.35* 12.21±0.41*	12.17±1.71 12.43±2.22*	**0.000** **0.000**
C-reactive protein _(mg/L)_	92.22±21.35	69.55±8.19	107.17±8.09*	97.67±30.45	**0.042**

The mean hemoglobin concentration was significantly different among CT-scan groups. However, on the seventh day of admission, it increased in the order of lung involvement scale from subtle to severe. C-reactive protein concentration was also significantly different among CT-scan groups. Data were expressed as Mean±SE. *Significantly different compared to the subtle group.

In all patients, the comparison of routine hematological parameters showed a significant increase in WBC (*P*<0.05) and platelets (*P*<0.001) after a week. In contrast, a significant decrease was observed in hemoglobin concentration (*P*<0.001). Also, the NAC increased during clinical management (Table. 4). The amount of MCHV, MCH, MCHC, and RDW was unchanged.

**Table 4:** The mean of routine hematologic parameters, at admission time and after the first week

	**Day 0**	**Day 7**	**P-value**
LAC (× 10^9/L)	127.0±8.5	140.4±6.5	**0.16**
NAC (× 10^9/L)	643.2±41.6	741.4±44.6	**0.04**
EAC (× 10^9/L)	17.7±2.1	16.8±2.4	**0.45**
MAC (× 10^9/L)	40.5±3.4	42.27±2.8	**0.63**
WBC (× 10^9/L)	8.25±0.48	9.44±0.48*	**0.03**
Platelet (× 10^9/L)	202.60±9.47	254.13±10.21*	**0.000**
Hemoglobin (g/L)	11.79±0.21	11.16±0.20*	**0.000**

The Neutrophil absolute count (NAC), White blood cells (WBC) and platelets were increased after seven days of hospital admission (Day 7) compared to the time of admission (Day 0), while Hemoglobin concentration was decreased. Data are expressed as Mean ±SE.

*Significant difference compared to admission time.

**Fig. 1:**
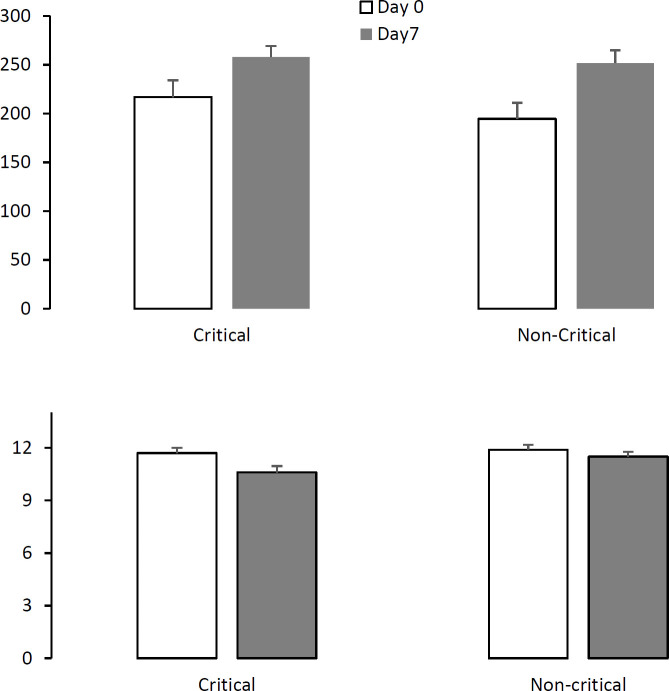
The mean of hemoglobin concentration and platelets counts during hospitalization in patients classified as critical and non-critical based on the severity of illness

In recovered or expired patients separately, the changes of hemoglobin and platelets concentration were similar to all patients. The comparison of complete blood count (CBC) parameters during admission did not change based on the severity of illness, unless for platelets that were increased significantly in both critical and non-critical patients on Day 7 (*P*<0.05) (Figure 1). Hemoglobin concentration was decreased only in the critical patients from 11.7±0.3 to 10.6±0.4 (*P*<0.05) (Figure 1).

Considering the image scoring, in the subtle group; hemoglobin was significantly decreased from 11.7±1.4 to 9.0± 1.1 (*P*<0.05). In the moderate group, WBC (from 7.2±0.6 to 9.8±0.7; *P*<0.005) platelets (from 209.3±16.7; *P*<0.005) and NAC (from 546.0± 50.6 to 768.9± 77.7; *P*<0.005) were increased during hospitalization. We also observed that in patients with more than 50% lung opacity, hemoglobin concentration and WBC were decreased while platelets count and NAC were increased significantly during admission (*P*<0.05). 

**Fig. 2:**
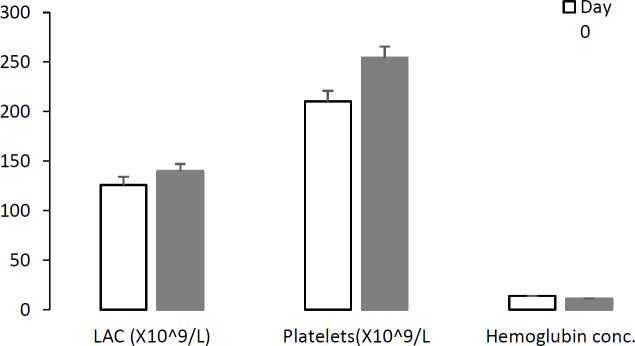
The mean hemoglobin concentration, platelet number, and leucocytes absolute count (LAC) during management in RT-PCR SARS-COV-2 positive patients

Comparison of main hematological parameters during a week in two groups of RT-PCR separately (positive and non-detected) showed that in RT-PCR SARS-COV-2 positive patients, hemoglobin concent-ration was decreased (*P*=0.002) while LAC and platelets were increased significantly (*P*=0.002) during hospitalization (Figure 2). In the non-detected RT-PCR group, the mean of platelets count was also increased significantly (*P*=0.013), but other parameters did not demonstrate any significant changes. The increase of platelets counts and decrease of hemoglobin concent-ration followed the same pattern for all patients previously reported in Table 4.

The mean of lactate dehydrogenase (LDH), C-reactive protein (CRP), and percent of opacity detected in CT-scan imaging (CT-scan opacity%) did not change significantly between alive and expired neither between critical and non-critical or positive and negative RT-PCR cases. Data are expressed as Mean ±SE

The mean of CRP was 93.3±5.3, and the mean LDH was 924.6±64.3 in recovered patients. The comparison of the mean CRP, LDH between alive and expired patients, critical and non-critical, and RT-PCR (positive and non-detected) patients did not show any difference (Table 5). Also, among image scoring groups, no change of CRP, LDH, or virus loads (Cycle Threshold values) was observed. 

**Fig. 3:**
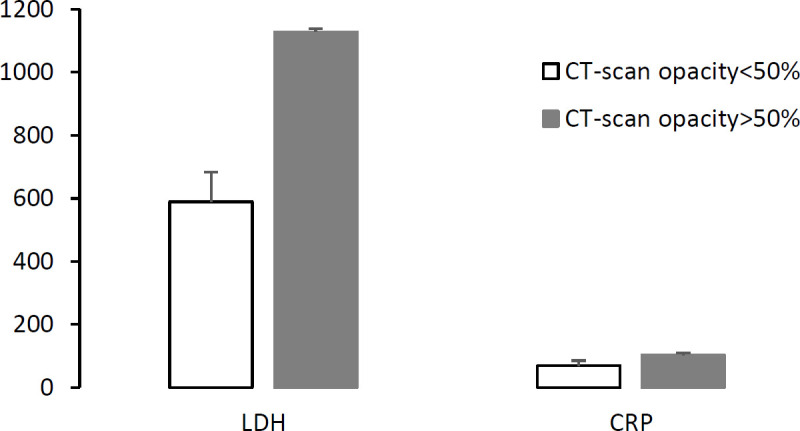
The mean of C-reactive protein (CRP) and lactate dehydrogenase (LDH) based on Ct-scan opacity of lung (<50% and >50%)

Finally, comparing patients with more than 50% lung opacity than those with less than 50% as diagnostic tool showed that both CRP and LDH were increased significantly in patients with more than 50% lung opacity in CT scan (*P*= 0.009 and *P*= 0.000 respectively) (Figure 3).

*Significant difference compared to admission time (*P*< 0.05)

*Significant difference compared to admission time (*P*< 0.05)

**Table 5:** The mean of LDH, CRP, and lung involvement (CT-scan opacity %) is based on the outcome, the severity of illness, and RT-PCR

		**LDH**	**CRP**	**CT-scan opacity%**
Outcome	Alive Expired	924.6±64.3 1288.1±211.2	93.3±5.3 114.1±13.9	**44.5±2.1** **44.3±8.4**
	P-value	0.06	0.23	**0.98**
Severity of illness	Critical Non-critical	1052.9±93.2 903.7±85.3	105.4±8.7 89.9±6.1	**48.3±4.5** **42.5±3.5**
	P-value	0.24	0.15	**0.32**
RT-PCR	Not detected Positive	853.4±178.2 1019±70.7	107.6±13.7 93.2±5.5	**44.0±6.1** **44.6±3.1**
	P-value	0.35	0.31	**0.95**

*Significant difference compared to CT-scan opacity of lung <50%. (*P*< 0.05)

## Discussion

The diagnosis of patients with COVID-19 requires laboratory molecular confirmation tests for SARS-COV-2 RNA (17, 19). Moreover, some laboratory tests are applied to assess COVID-19 severity, complications, and management (5). 

Based on a meta-analysis, most abnormal laboratory tests in the first week of COVID-19 infection showed an increase of CRP, LDH, and lymphopenia consistent with our findings (7, 8, 18). Similarly, the other studies from the SARS outbreak in 2003 demonstrated that the viral disease SARS might be associated with lymphopenia, leukopenia, thrombocytopenia, and elevated levels of LDH, ALT, AST, and CK (24, 25).

In our study population, 8.7% were expired, and 36.2% were diagnosed as critical COVID-19 cases. Based on the Chinese CDC clinical scoring for SARS-CoV-2 infection, seventy-nine (60.8%) were classified as mild, 42 (32.3%) as severe, and nine patients (6.9%) as critical (26). 

In this study comparison of routine hematological indexes showed a significant increase of WBC (*P*<0.001) and platelets (*P*<0.001) after a week. In contrast, a significant decrease was reported in hemoglobin concentration (*P*<0.001). This increase of platelets was also significant comparing critical patients (severe) with non-critical ones (subtle to moderate). Based on research in medical databases, the hemoglobin and platelet levels were found to be significantly different in severe cases compared to those with milder disease, in which the analysis entailed the estimation of the weighted mean difference of −7.1 g/L; 95% CI= −8.3 to−5.9 g/L between the two groups (23). 

We also found a considerable correlation between outcome and clinical severity (*P*=0.000). A great number of published articles and meta-analyses have indicated that higher total leukocyte count and neutrophils are associated with severe COVID-19 cases and worse prognosis (12, 19, 27, 28). 

Other studies discussed the pathophysiology role of neutrophils in COVID-19 inflammation. Research revealed upregulation of neutrophil genes and chemokines. Different inflammatory genes consisting of TNFR, IL-8, CXCR1, CXCR2, ADAM10, GPR84, MME, ANPEP, and LAP3 might be targets in COVID-19 inflammation in severe critical cases (29).

In our study, hemoglobin concentration and CRP used as biomarkers showed significant changes among CT-scan scoring groups. Also, WBC (from 7.2±0.6 to 9.8±0.7; *P*<0.005), platelets (from 209.3±16.7; *P*<0.005) and NAC were increased (from 546.0±50.6 to 768.9± 77.7;* P*<0.005) during hospitalization. 

The circulating biomarkers, including CRP, are known to interact with erythrocytes and platelets pathology, resulting in severe vasculopathy in critical COVID-19 cases. Consistent with our findings, the structural pathologic changes in platelets and RBCs may play a role in thrombotic microangiopathy as well as ground-glass opacities in the lung (30, 31). We found that LAC increased significantly during COVID-19 recovery. Similarly, researchers reported a significant increase in LAC in the second week of illness during the recovery (32, 33). Furthermore, Kazancioglu *et al.* mentioned that leukocytes and neutrophils were increased while hemoglobin and hematocrit levels were decreased in critical COVID-19 patients at the end of treatment (11). 

Interestingly in cases with more than 50% lung-opacity, hemoglobin concentration and WBC were decreased while platelets count and NAC were increased significantly during a week of recovery (*P*<0.05). Moreover, the biomarkers CRP and LDH were increased notably in the cases with more than 50% lung opacity in lung tomography (Figure 3).

The above findings are consistent with the Yang et al. study that reported a higher percentage of neutrophils, CRP, and procalcitonin in the severe CT-Scan group and recommended optimal inflammation load score to identify severe COVID-19 with acceptable diagnostic sensitivity and specificity (34). In addition, Francone* et al. *emphasized that CT score is highly correlated with laboratory findings and COVID-19 severity (35).

There was no clear correlation between NP/OP viral loads (Cycle Threshold values), clinical status, and lung image severity (CTSS) with biochemical markers of CRP, LDH, and routine hematological parameters in our study. However, hemoglobin levels in RT-PCR detected group decreased significantly (*P*=0.002) after a week.

Hasanoglu* et al. *performed a similar study on sixty COVID- 19 patients. They found that in patients with bilateral ground-glass opacity in chest CT, the viral loads were significantly lower when compared with patients with unilateral ground-glass opacity or normal chest CT-scan. A significant decrease in viral load was observed with increasing disease severity. In correlation of SARS-COV-2 viral load with laboratory findings, there was no difference between PCR-detected and non-detected cases. They also showed a significant positive correlation of LAC with upper respiratory viral load (*P*=0.0469) and among the hematological parameters (16). Considering respiratory viral dynamics, low OP/NP viral load in critical COVID-19 patients can be rated to the late admission to hospital. Also, the duration of viral shedding is longer in men than women and higher in elderly patients (36, 37).

## Study Limitations

First, we accomplished a retrospective study of only a proportion of SARS-COV 2 patients admitted to a hospital. Secondly, dynamic observation of the SARS-COV-2 virus was not available. Dynamic observation is more representative than single Real-time PCR CT values for quantification of viral load. Finally, bronchoalveolar lavage tests could not be carried out commonly. We could only conduct the molecular test using an upper respiratory swab sample to reflect the patient’s SARS-COV-2 viral load, a heterogeneous sample. 

## Conclusion

The recent reports of SARS-COV2 variants of concern (VOC) from the United Kingdom, South Africa, Brazil, and India have shown increased concern for the severity features of infection , transmissibility, and clinical presentations (38). Our findings suggest that a combination of routine biochemical markers and hematologic parameters (including hemoglobin, neutrophil count, and platelets) with lung tomography score might be beneficial for making diagnosis, assessment of the severity of the disease, and prognosis in the hospitalized cases. LAC was increased during COVID-19 recovery, suggesting additional research to determine if these lymphocytes are protective or immunopathogenic in COVID-19 patients.

## Funding

There are no financial disclosures to report on any of the authors.

## Authors Contribution

The authors confirm contribution to the article as follows: study conceptualization and design: A. Javadi; Laboratory analysis and technical support: A.javadi, H. Vosough; data collection: M. Gheidi Shahran, F.Sedaghati; Data analysis and interpretation of results: M.Shamsi-Meymandi, A.Javadi, H. Soleimantabar; Draft Writing- Review & Editing: A. Javadi, M. Shamsi-Meymandi, S. Dabiri, B.Dabiri, M. Hashemi-Bahremani.

## Conflict of Interest

The authors declared no conflict of interest.
